# The Important Role of Intermuscular Adipose Tissue on Metabolic Changes Interconnecting Obesity, Ageing and Exercise: A Systematic Review

**DOI:** 10.17925/EE.2023.19.1.54

**Published:** 2023-05-17

**Authors:** I Gusti Putu Suka Aryana, Ivana Beatrice Paulus, Sanjay Kalra, Dian Daniella, Raden Ayu Tuty Kuswardhani, Ketut Suastika, Sony Wibisono

**Affiliations:** 1. Geriatric Division, Department of Internal Medicine, Faculty of Medicine, Udayana University/Prof. I Goesti Ngoerah Gde Ngoerah Teaching Hospital, Denpasar, Bali, Indonesia; 2. Wangaya General Hospital, Denpasar, Bali, Indonesia; 3. Bharti Hospital, Karnal, India; 4. Department of Research, Chandigarh University, Chandigarh, India; 5. Department of Internal Medicine, Faculty of Medicine, Udayana University/ I Goesti Ngoerah Gde Ngoerah Teaching Hospital, Bali, Denpasar, Indonesia; 6. Geriatric Division, Department of Internal Medicine, Faculty of Medicine, Udayana University/ I Goesti Ngoerah Gde Ngoerah Teaching Hospital, Denpasar, Bali, Indonesia; 7. Division of Endocrinology and Metabolism, Department of Internal Medicin, Faculty of Medicine, Udayana University/ I Goesti Ngoerah Gde Ngoerah Teaching Hospital, Denpasar, Bali, Indonesia; 8. Division of Endocrinology and Metabolism, Airlangga University, Soetomo Teaching Hospital, Surabaya, Indonesia

**Keywords:** Ageing, exercise, intermuscular adipose tissue, metabolic changes, obesity, sarcopenic obesity

## Abstract

As age increases, adipose tissue infiltrates muscle tissue and leads to sarcopenia. When excessive accumulation of adipose tissue accompanied progressive decrease in lean body mass especially visceral fat, termed as sarcopenic obesity (SO) and related metabolic intermuscular adipose tissue (IMAT) is an ectopic tissue found between muscle groups, and is distinct from subcutaneous adipose tissue. Until now, the association between IMAT and metabolic health was not understood. This study is the first systematic review assessing the association between IMAT and metabolic health. The PubMed, Science Direct and Cochrane databases were searched for studies reporting IMAT and metabolic risk. The descriptions of the extracted data are guided by the Preferred Reporting Items for Systematic Reviews (PRISMA) statement with a Grading of Recommendations Assessment, Development and Evaluation approach. This study is registered at PROSPERO (identifier: CRD42022337518). Six studies were pooled and reviewed using critical appraisal by the Newcastle Ottawa Scale and Centre for Evidence-Based Medicine checklist. Two clinical trials and four observational trials were included. Our results reveal that IMAT is associated with metabolic risk, especially in older adults and patients with obesity. However, in a person with abdominal obesity, VAT has a more significant role in metabolic risk than IMAT. The largest decrease in IMAT was achieved by combining aerobic with resistance training.

Obesity is related to an increased risk of metabolic disease.^1^ It is well established that excessive body fat is related to increased morbidity and mortality and is associated with inflammation and endothelial damage.^2,3^ With age, fat infiltration occurs in the muscle, leading to sarcopenia. The association of the excessive accumulation of adipose tissue with a progressive decrease in lean body mass, especially visceral fat, is termed sarcopenic obesity (SO).^4,5^

Adipose tissue is classified into subcutaneous adipose tissue (SAT) and ectopic adipose tissue. Ectopic adipose tissue is pathological. Intermuscular adipose tissue (IMAT) is one of ectopic adipose tissue beside visceral adipose tissue. IMAT is directly related to increasing risk of insulin resistance, subclinical atherosclerosis, metabolic syndrome and cardiovascular disease.^6–10^ Adipokines such as monocyte chemoattractant protein-1 (MCP-1), leptin and adiponectin are secreted from IMAT and play a critical role in obesity-related insulin resistance, altered lipid metabolism and inflammatory response.^11,12^ IMAT accretion is mostly described in degenerative muscular disorders associated with age; however, it has also been seen in chronic conditions associated with a decline in physical activity due to reduced muscle contractile function.^13^ Exercise training may lessen the overall amount of fat deposited in tissues and organs and of adipose tissue, including IMAT.^14^

Until now, studies associating IMAT and metabolic change is not yet clear due to inconsistent result of studies. This study is the first systematic review to investigate the association between IMAT and metabolic syndrome.

**Table 1:** Method of searching^15–17^

Database	Keyword	Hits
Pubmed^15^	(("Intermuscular"[All Fields] AND "adipose tissue"[MeSH Terms]) OR "intermuscular adipose tissue"[Title/Abstract] OR "IMAT"[Title/Abstract]) AND ("metabolic syndrome"[MeSH Terms] OR "mets"[Title/Abstract] OR "metabolic syndrome"[Title/ Abstract]) Translations Adipose Tissue[MeSH Terms]: "adipose tissue"[MeSH Terms] Metabolic syndrome[MeSH Terms]: "metabolic syndrome"[MeSH Terms] Warnings (((((Intermuscular Adipose Tissue[MeSH Terms])) OR (Intermuscular Adipose Tissue[Title/ Abstract])) OR (IMAT[Title/Abstract]))) AND (((((Metabolic syndrome[MeSH Terms]) OR (Mets[MeSH Terms])) OR (mets[Title/Abstract])) OR (metabolic syndrome[Title/Abstract])))	370
CENTRAL^16^	(Intermuscular Adipose Tissue) AND (Metabolic syndrome)	20
Science Direct^17^	(Intermuscular Adipose Tissue) AND (Metabolic syndrome)	857

## Methods

### Eligibility criteria

Eligible publications were screened independently by the authors. Inclusion criteria were articles 1) written in the English language, 2) published in 2012 or later and 3) reporting a clinical trial or observational study. Letters, viewpoints and reviews that provided further insight into IMAT and metabolic parameters were excluded. Included studies reported the association between IMAT and metabolic syndrome.

Cardiovascular and metabolic parameters, namely MCP-1 and insulin sensitivity index (ISI) and metabolic syndrome risk, were assessed. The Japanese criteria for metabolic syndrome included a special criteria for high waist circumference of ≥85 cm in men and ≥90 cm in women but not high body mass index (BMI ≥25 kg/m^2^) due to the unique anthropometricy seen in the Japanese population.

### Search strategy

The literature review was carried out using the keywords "(Intermuscular Adipose Tissue) AND (metabolic syndrome)" in the PubMed, Science Direct and Cochrane Central Register of Controlled Trials databases (*Table 1*).^15–17^

### Data extraction and assessment

All references were reviewed using the critical appraisal Newcastle– Ottawa Scale^18^ and the Centre for Evidence-Based Medicine checklist.^19^ Each author independently reviewed all titles and abstracts to identify irrelevant studies. The titles and abstracts of articles were used to select those eligible for the first screening based on the established inclusion and exclusion criteria and were screened for articles reporting IMAT and metabolic syndrome. Articles that passed the first screening were checked in full to identify those relevant to the relationship between IMAT and metabolic syndrome. Articles that did not meet the inclusion criteria were eliminated. The authors' names, year of publication, sampling period, study location, sample size, study design, age range of participants, duration of intervention and outcomes were collected from the chosen studies. Then, each author independently scored and assessed the risk of bias of all included articles using a checklist based on the Newcastle– Ottawa Scale for observational studies and the Cochrane Handbook for Systematic Reviews of Interventions. Results were compared, and any controversies surrounding any included or excluded paper were resolved by a discussion. Potentially eligible manuscripts were exported. The descriptions of the extracted data were guided by the Preferred Reporting Items for Systematic Reviews statement. The level of evidence was applied as per Grading of Recommendations Assessment, Development and Evaluation (GRADE) criteria and reported.^20^ This study is registered in the PROSPERO database (PROSPERO identifier: CRD42022337518).

## Results

### Study characteristics

**Figure 1:**
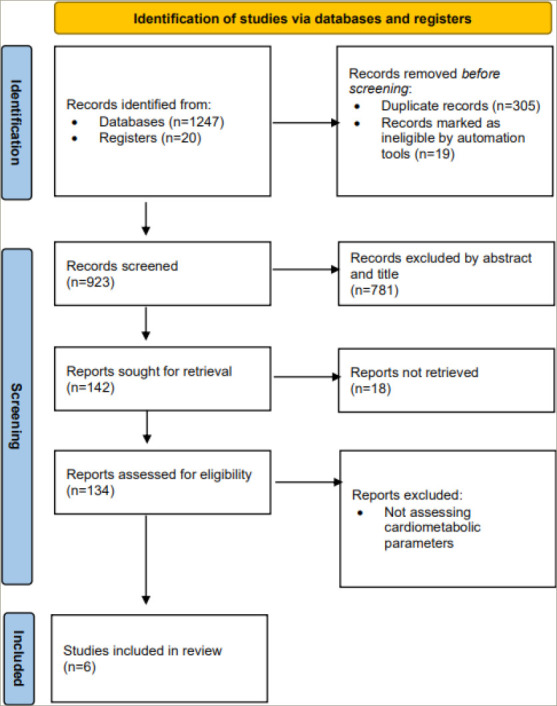
PRISMA flowchart of search strategy

The literature search results and study selection process are presented in *Figure 1*. Before screening, 305 duplicate records and 19 records marked ineligible by automation tools were removed. Then, 124 articles were excluded because they did not assess metabolic parameters or they were animal studies and viewpoints and review studies. Ten potential articles were chosen to undergo full assessment for eligibility in the current review. Finally, the systematic review, included six publications on the impact of IMAT on metabolic parameters: four observational trials and two clinical trials. In *Table 2* and *Table 3*, an overview and critical evaluation of studies that qualified for the systematic review are described. The level of evidence by GRADE was low to moderate due to the suboptimal quality of research.^21–26^

## Discussion

### Adults who are obese

In a cross-sectional study performed by Ham et al. among 77 Korean women who are obese, MCP-1 level positively correlated with the low-density muscle (r=0.355; p=0.002) and IMAT (r=0.483 p<0.001); the correlation of MCP-1 to IMAT remained significant when adjusted for VAT, SAT and high-density muscle in a multivariate analysis (β=0.433, p=0.001).^23^ Low-density muscle were considered as areas that had attenuation values between 0 and 34 HU, which indicates fat-rich muscle, while the areas that had attenuation values between 35 and 100 were regarded as high-density muscle, which indicates normal muscle.

MCP-1 is one of the major adipokines secreted by adipose macrophages as well as a proinflammatory cytokine that plays a role in metabolic dysfunction.^27^ It helps recruit immune cells, such as macrophages and T cells, in the extracellular signal-regulated kinase pathway to increase insulin resistance.^6,28,29^ Leptin is an adipokine derived from adipose tissue that contributes to inflammatory processes.^30,31^ In one study, leptin messenger RNA (mRNA) was expressed more in older patients than in young patients.^32^ Metabolically healthy obese individuals are also associated with better adipose tissue composition and capacity.^33^ Haam et al. showed that MCP-1 levels were associated with increased levels of Homeostatic Model Assessment for Insulin Resistance (HOMA-I R) and insulin.^23^ However, the association between leptin levels and IMAT was found to be nonsignificant after adjustments for VAT, SAT and high-density muscle (β=-0.051; p=0.686).

### Middle-aged adults

Bang et al. studied 166 middle-aged and older Japanese adults without exercise habits or severe metabolic disease (mean age 68.9 ± 5.5 years; 53 men, 113 women).^21^ Thigh muscle and abdominal cross-sectional area were measured by magnetic resonance imaging (MRI). This study reported a low prevalence of obesity according to BMI (1.3% of patients has a BMI of >30) but a high prevalence of abdominal obesity (27.1%) (mean WC 81.9 ± 9.6 cm).

The result shows that metabolic syndrome risk factors were significantly associated in the highest quartile of IMAT, with a cut-off above 5.5 cm^2^, compared with the lowest quartile, with a cut-off below 2.5 cm^2^ (p <0.001). IMAT quartiles were significantly associated with the frequency of abdominal obesity (p<0.001) and hyperglycaemia (p<0.01). Multiple regression showed that the association between IMAT accumulation and the number of metabolic syndrome risk factors persisted after adjustment for BMI (β=0.204; R^2^=0.325; p<0.01) and VAT (β=0.177; R^2^=0.349; p<0.05). IMAT, especially thigh measurement, correlated with metabolic syndrome components as defined above.

Several studies reported a similar result and stated, based on Spearman's correlation, that an increase in IMAT in paraspinosus correlated with metabolic syndrome and a decrease in muscle attenuation is linked with a 1.2-fold increase in metabolic syndrome risk among middle-age individuals.^34–36^ Goodpaster et al. reported older adults with normal weight and higher-thigh IMAT had a higher risk of metabolic syndrome than participants with normal weight and lower-thigh IMAT.^34,37^ One hypothesis to describe this phenomenon involves the concept of metabolically obese, normal-weight individual.^2^ The regional adipose tissue may determine glucose regulation related to metabolic syndrome.^37^ The relationship between muscle, fat and insulin may be explained by increased intramyocellular lipid (IMCL)-associated insulin resistance and be related to low oxidative capacity.^38^ Many previous studies have reported that oxidative capacity in skeletal muscle has a role in IMCL accumulation and insulin resistance.^39^ Many previous studies have reported that oxidative capacity in skeletal muscle has a role in IMCL accumulation and insulin resistance.^38–41^ The uptake of free fatty acid in muscle by fatty-acid transporters, such as plasma membrane-associated fatty acid-binding protein (FABPpm), stimulates fatty-acid transport and increases the oxidative capacity of fatty acids pathways.^42^ This may explain why IMAT is related to metabolic syndrome, as high levels of fat deposits in muscle cells are related to low expression of FABPpm.^43,44^

**Table 2:** Overview of eligible studies^21–26^

Author	Study design	Participants	Mean age (years) ± SD	Methods	Outcomes
**Haam et al.** ^23^	Observational, crosssectional	77 obese Korean women	41.7 ± 10.9	Investigated the association between adipose parameters such as VAT, SAT, IMAT and MCP-1. The adipose parameters were measured using CT scans, while biochemical marker were assessed using ELISA	Bivariate analysis showed a statistically significant correlation between MCP-1 and IMAT (r=0.483; p<0.001)
**Goss and Gower** ^22^	Observational, crosssectional	97 healthy post-menopausal women aged 45–60 years	50.8 ± 2.8	Identify the association between insulin and thigh adipose including IMAT. CT scans and dual-energy X-ray absorptiometry were used to assess thigh adipose tissue, while while metabolic marker asses insulin sensitivity. This study has aimed to identify the association between insulin and thigh adipose including IMAT.	ISI was negatively associated with thigh IMAT (r=-0.54). Thigh IMAT strongly correlated with intraabdominal adipose tissue (r=0.69)
**Bang et al.** ^21^	Observational study	166 middle-aged and older Japanese adults	68.9 ± 5.5	Investigated the relationship between thigh IMAT and metabolic syndrome. IMAT was examined using MRI and metabolic syndrome was defined according to Japanese diagnosis criteria.Patients were divided into quartiles according to IMAT (Q1: below 2.7 cm^2^; Q2: 2.7–3.7 cm^2^; Q3: 3.8–5.5 cm^2^; Q4: above 5.5 cm^2^)	Metabolic syndrome risk factors were significantly associated in higher quartiles than in lower (p<0.001). Multivariate analysis showed association between IMAT accumulation and several metabolic syndrome risk factors, BMI and VAT (p<0.05)
**Lim et al.** ^24^	Observational study	187 community-dwelling elderly	67.8 ± 7.8	Examined the association between IMAT, MCP-1 and several performance parameters. Muscle strength and physical performance were assessed. While blood inflammatory markers were measured by MCP-1, IMAT was measured by MRI	Pearson analysis shows MCP-1 and IMAT statistically significantly correlate (r=0.286; p<0.05). IMAT negatively correlated with grip strength, knee extension strength and gait speed (r=-0.244, p=0.001; r=-0.205, p=0.005; r=-0.170, p=0.020, respectively)
**Yaskolka Meir et al.** ^25^	RCT	278 participants with abdominal obesity	47.8 ± 9.3	Investigated the effect of 18 months of lifestyle intervention on IMAT and TMA. IMAT, TMA and body composition were measured using MRI	The IMAT and TMA were comparable to the nonintervention group's, IMAT and TMA and they correlated with modest weight reduction (p<0.001) IMAT change did not remain independently associated with decreasedabdominal subdepots or improved cardiometabolic parameters afteradjustments
**Waters et al.** ^26^	RCT	160 obese older adults	70.0 ± 5.0	Investigated the effects of aerobic, resistance or combination exercise on total adipose tissue and IMAT. This study tested baseline and 1-year MRI measures of total adipose tissue (AT) including IMAT.	Changes in IMAT and VAT were linked with PPT (r=-0.28 and r=-0.39, respectively), VO_2 peak_ (r=-0.49 and r=-0.52, respectively), gait speed (r=-0.25 and r=0.36, respectively) and ISI (r=0.49 and r=-0.52, respectively) (for all, p=0.05)

*AT = adipose tissue; BMI = body mass index; CT = computed tomography; ELISA = enzyme-linked immunosorbent assay; IMAT = intermuscular adipose tissue; ISI = insulin sensitivity index; MCP-1 = monocyte chemoattractant protein-1; MRI = magnetic resonance imaging; PPT = physical performance test; RCT = randomized controlled trial; SAT = subcutaneous adipose tissue; TMA = thigh muscle area; VAT = visceral adipose tissue.*

**Table 3:** Critical appraisal of eligible studies^21–26^

Author	Design	Selection	Comparability	Outcome	
Lim et al.^24^	Cross-sectional study	***	*	***	
Haam et al.^23^	Cross-sectional study	**	*	***	
Goss and Gower^22^	Cross-sectional study	****	*	***	
Bang et al.^21^	Cross-sectional study	****	*	***	
		Validity	Importance	Applicability	
Yaskolka Meir et al.^25^	RCT	(+)	(+)	(+)	
Waters et al.^26^	RCT	(+)	(+)	(+)	

-= not fulfilled the criteria; + = positive and fulfilled the criteria; * = one point; RCT = randomized controlled trial.

However, the study by Bang et al. did not directly assess the association between IMAT and FABPpm.^21^

## Older adults

Lim et al. studied 187 community-dwelling older participants, who were recruited and classified into four subgroups: normal, obese, sarcopenia and SO.^24^ They evaluated the association between IMAT and several metabolic and physical performance measures. Anthropometry was documented by MRI or dual-energy X-ray absorptiometry, MCP-1 was measured in venous blood samples and grip strength was recorded using a hydraulic hand dynamometer. IMAT was reported as the ratio of IMAT volume to total volume of the imaged thigh.

Of these 187 participants, 102 (54.6%) were obese, 42 (22.5%) were sarcopenic and 17 (9.1%) had SO. This study revealed that IMAT was higher in the SO and obese groups (M=0.0335 ± 0.0178 and M=0.0274 ± 0.0128, respectively). MCP-1 was significantly associated with IMAT according to Pearson's correlation (r=0.286; p<0.05). Grip strength, knee extension strength and gait speed were negatively associated with IMAT (r=-0.244, p=0.001; r=-0.205, p=0.005; r=-0.170, p=0.020, respectively).^24^

The European Working Group on Sarcopenia in Older People defines sarcopenia as a syndrome with progressive and generalized loss of skeletal muscle mass and strength.^45^ During the ageing process, muscle is depleted and replaced by fat. SO arises from the excessive accumulation of adipose tissue, especially visceral fat, and a decrease in lean body mass.^46^ IMAT is an ectopic fat, and its accumulation promotes muscle lipid excess via interstitial free fatty acids. IMAT has been postulated to be associated with age and fatty infiltration into the skeletal muscle. It may cause cytokine release which related to metabolic changes.^47^ IMAT mRNA accumulation genes regulate lipolysis, insulin sensitivity and inflammatory cytokines such as MCP-1.^6,7^ Perkisas et al. have also reported a negative correlation between IMAT, hand grip strength and functionality (short physical performance battery).^48^

## Post-menopausal adults

Goss and Gower studied 97 healthy, early post-menopausal women and assessed the relationship between insulin sensitivity and the subcompartments of thigh adipose tissue.^22^ In this study, thigh IMAT and intra-abdominal adipose tissue (IAAT) were negatively associated with ISI. The highest correlation coefficients were between abdominal SAT and thigh SAT (r=0.73), IAAT and thigh IMAT (r=0.69), and IAAT and abdominal SAT (r=0.69). The correlation between ISI and thigh IMAT was negatively correlated in simple (r= -0.51; p<0.001) but not in multiple linear regression (standardized β=0.05 ; p=0.72). After adjusting for high IAAT, tight IMAT and ISI were found to be negatively correlated (standardized β=-0.8; p=0.01) .^22^

Menopause predisposes women to weight gain and fat accumulation, especially visceral fat.^40,49,50^ During this phase circulating estradiol declined by up to 10 pg/mL. Low oestrogen, specially E2, predispose menopausel women to cardiovascular event. Oestrogens is majority syntized in ovaries by using low-density lipoprotein-cholesterol. During menopause, oestrogen levels decrease, leading to increased blood low-density lipoprotein-cholesterol levels and enhanced cardiovascular disease risk. Menopausal women can have lipid disturbance and a high prevalence of abdominal obesity.^51^

## Intermuscular adipose tissue-targeted evidence

A randomized controlled trial by Waters et al. studied the effect of 26 weeks of exercise training on the metabolic function and fat composition in 160 older adults with obesity.^26^ Participants were assigned to one of four groups: control, aerobic exercise (AEX), resistance exercise (REX) or combination exercise (COMB). At the end of the study, parameter changes were estimated using MRI for IMAT and VAT and oral gluce tstolerance tests for ISI, and physical function was assessed using Modified Physical Performance Test (PPT), peak oxygen consumption (VO_2peak_) and gait speed. Knee strength was measured by dynamometry. A greater decrease in IMAT and VAT experience was noted in the COMB group than in the AEX and REX groups (IMAT -41%, -28% and -23% for COMB, AEX and REX groups, respectively; VAT -36%, -19% and 21%, respectively; p=0.003 to 0.01); after intervention, ISI increased by 86%, 50% and 39% in the AEX, COMB and REX group, respecitvely (p=0.05 to 0.3), and PPT was higher in COMB group. IMAT significantly correlated with VO_2peak_ (r=-0.49; p=0.45), gait speed (r=-0.25; p=0.04), ISI (r=-0.49; p<0.001) and PPT score (r=-0.28; p=0.02).^26^

Insulin improves physical function by enhancing insulin-mediated blood flow and muscle protein synthesis and may improve myocellular energetics.^7,48,49^ IMAT has a negative association with muscle function due to its effect on glucose uptake via the Janus kinase-signal transducer and activator of transcription and mitogen-activated protein kinase pathways.^6,52^ Physiologically, insulin inhibits lipolysis, while insulin resistance may induce the local release of free fatty acids into the interstitial fluid surrounding muscle by increasing myotube 1,2-diacylglycerol content.^12,53^ Previous studies have revealed an association between IMAT and insulin sensitivity.^6,54,55^ Similarly, the study by Waters et al. found that IMAT was associated with ISI (r=-0.49; p<0.001).^26^

A study by Meir et al.^25^ involved 278 sedentary participants with abdominal obesity and randomized them into groups in two phases. First, participants were organized into two diet groups: a low-fat diet or a Mediterranean/low-carbohydrate diet; second, participants were divided into groups with or without an add-on moderate physical activity intervention after 6 months. Physical activity was aerobic in over 80% of participants assigned to the physical activity intervention. After 18 months, changes in IMAT were found to not be directly related to the composition of visceral fat. Physical activity intensity levels were also measured using metabolic equivalent of task (MET, that is, an indication of physical activity intensity levels) units, defined as the ratio of work metabolic rate to the standard resting metabolic rate. MET level can range from 0.9 (sleeping) up to 18 METs (fast running).

In addition, there was a significant change in MET between the physical activity and no physical activity groups (p=0.009). IMAT loss was associated with declining triglycerides (β=0.152), cholesterol (β=0.174), HOMA-I R (β=0.175), glycated haemoglobin (β=0.165), leptin (β=0.19) (p<0.05 for all) and with high-density lipoprotein-cholesterol (β=-0.195; p<0.01) in an age-and sex-adjusted model. Further adjustments for WC, VAT and BMI changes remarkably attenuated IMAT associations.^25^ During ageing, lipids are deposited and infiltrated into muscle tissues, a process called myosteatosis. Ectopic fat negatively correlates with muscle mass, strength and mobility and may affect metabolism.^48,56^ IMAT contributes to sarcopenia by increasing the storage of lipids in adipocytes underneath the deep fascia of muscle, especially in older adults.^57,58^

Our study is the first to systematically review of IMAT-related evidence to metabolic changes. Limited research has been conducted so far, and the included studies vary in outcomes, intervention and study design. There is no head-to-head comparison between each study.

The level of evidence by GRADE has been graded low to moderate due to the high risk of bias, inconsistency and uncertainty; therefore, the findings should be interpreted as low quality. More randomized controlled trials of IMAT and metabolic health are needed to provide better information and improve the quality of the review. It may also be meaningful to use a combination of VAT and IMAT as a marker of metabolic health and as a target for intervention.

## Future directions and conclusions

IMAT is associated with metabolic syndrome risk, especially in older and adults who are obese. However, in people with abdominal obesity, VAT has a more significant role in metabolic syndrome than IMAT. IMAT decreased the most in participants that underwent combined aerobic and resistance exercise. We suggest the IMAT is screened and addressed in patients susceptible to metabolic syndrome to reduce the rates of metabolic events.
